# PolyADP-Ribosylation Is Required for Pronuclear Fusion during Postfertilization in Mice

**DOI:** 10.1371/journal.pone.0012526

**Published:** 2010-09-02

**Authors:** Tomoharu Osada, Hideki Ogino, Toshiaki Hino, Sachiyo Ichinose, Kenji Nakamura, Akira Omori, Toshiaki Noce, Mitsuko Masutani

**Affiliations:** 1 Advanced Medical Science Research Center, Mitsubishi Chemical Medience Corporation, Minato-ku, Tokyo, Japan; 2 Department of Regenerative and Developmental Biology, Mitsubishi Kagaku Institute of Life Sciences (MITILS), Machida, Tokyo, Japan; 3 Biochemistry Division, National Cancer Research Institute, Chuo-ku, Tokyo, Japan; 4 ADP-ribosylation in Oncology Project, National Cancer Research Institute, Chuo-ku, Tokyo, Japan; 5 Mouse Genome Technology Laboratory, Mitsubishi Kagaku Institute for Life Sciences (MITILS), Machida, Tokyo, Japan; 6 Bio-molecular Structure Analysis Laboratory, Mitsubishi Kagaku Institute for Life Sciences (MITILS), Machida, Tokyo, Japan; Institute of Zoology, Chinese Academy of Sciences, China

## Abstract

**Background:**

During fertilization, pronuclear envelope breakdown (PNEB) is followed by the mingling of male and female genomes. Dynamic chromatin and protein rearrangements require posttranslational modification (PTM) for the postfertilization development.

**Methodology/Principal Findings:**

Inhibition of poly(ADP-ribose) polymerase activity (PARylation) by either PJ-34 or 5-AIQ resulted in developmental arrest of fertilized embryos at the PNEB. PARylation inhibition affects spindle bundle formation and phosphorylation of Erk molecules of metaphase II (MII) unfertilized oocytes. We found a frequent appearance of multiple pronuclei (PN) in the PARylation-inhibited embryos, suggesting defective polymerization of tubulins. Attenuated phosphorylation of lamin A/C by PARylation was detected in the PARylation-inhibited embryos at PNEB. This was associated with sustained localization of heterodomain protein 1 (HP1) at the PN of the one-cell embryos arrested by PARylation inhibition.

**Conclusions/Significance:**

Our findings indicate that PARylation is required for pronuclear fusion during postfertilization processes. These data further suggest that PARylation regulates protein dynamics essential for the beginning of mouse zygotic development. PARylation and its involving signal-pathways may represent potential targets as contraceptives.

## Introduction

Fertilization comprises a series of biological steps beginning with the recognition between the egg and sperm cells and ending at the mingling of genetic materials of these two cells [1]. Previous studies have elucidated the behavior of various cell organelles and proteins within the egg during fertilization [2]. In humans, arrest of fertilized eggs at the pronuclear (PN) stage is commonly observed after *in vitro* fertilization (IVF) or intracytoplasmic sperm injection (ICSI) [3]. We know little about the molecular mechanisms of the pronuclear envelope breakdown (PNEB) and the mingling of male and female genomes. Since zygotic genes are largely expressed only after the first cleavage of embryos [4], it is most likely that the posttranslational modification (PTM) of maternal proteins plays central regulatory roles before and during the PNEB.

A wealth of study has reported the dynamic PTMs of nuclear proteins during the first cell-cycle of mouse development. Phosphorylation transmits intracellular signals into nuclear proteins, which mainly drives progression of the first cell-cycle [5]. Like in carcinogenesis and other cellular processes, chromatin modification systems including histone acetylation and methylation in early embryos are involved in the gene expression regulation mediated by remodeling of chromatin structure [6]. Chromatin modifications are different between parental chromatins at the one-cell embryo [7]. Although biological significance of the PTM is elusive during postfertilization development, it is acceptable that the maternal PTM would regulate zygotic gene activation at the 2-cell stage embryos [8].

To understand the molecular machinery essential for the postfertilization events, we studied the effects of reagents that affect poly(ADP-ribosylation) (PARylation). Poly(ADP-ribose) polymerase (Parp) is known to contribute to DNA repair, transcription, and spindle assembly by transferring negatively charged poly(ADP-ribose) polymers (PAR) to acceptor proteins [9], [10]. While the mice lacking Parp1, the most abundant PARP, are viable and fertile [11], those lacking both Parp1 and Parp 2 die at the onset of gastrulation [12]. PARylation is also regulated by poly(ADP-ribose) glycohydrolase (Parg), which cleaves ribosyl-ribose linkages of ADP-ribose polymer. Mice lacking the *Parg* gene are lethal during cleavage-stage of mouse embryogenesis, with accumulation of ADP-ribose polymers [13]. These data suggest that the PARylation contributes to the early stages of mouse embryogenesis. Recent studies elucidated that PARylation system is regulated by Parp family genes, 17 of which have been identified so far [10]. We addressed the role of total PARylation reactions catalyzed by members of Parp family during fertilization process, utilizing PARP inhibitors. In the case of Parp knockout animals, we are not able to avoid compensatory effects of other Parp family members. The use of PARP inhibitors could enable us to examine the effects of blocking whole PARylation reactions. These data will elucidate biological windows for the dissection of the complex PARylation system during mouse embryogenesis.

## Results

### Levels of Parp1, ADP-ribose polymer, Parg, and Parp-family gene expression in MII oocytes and postfertilized embryogenesis

**Figure 1:**
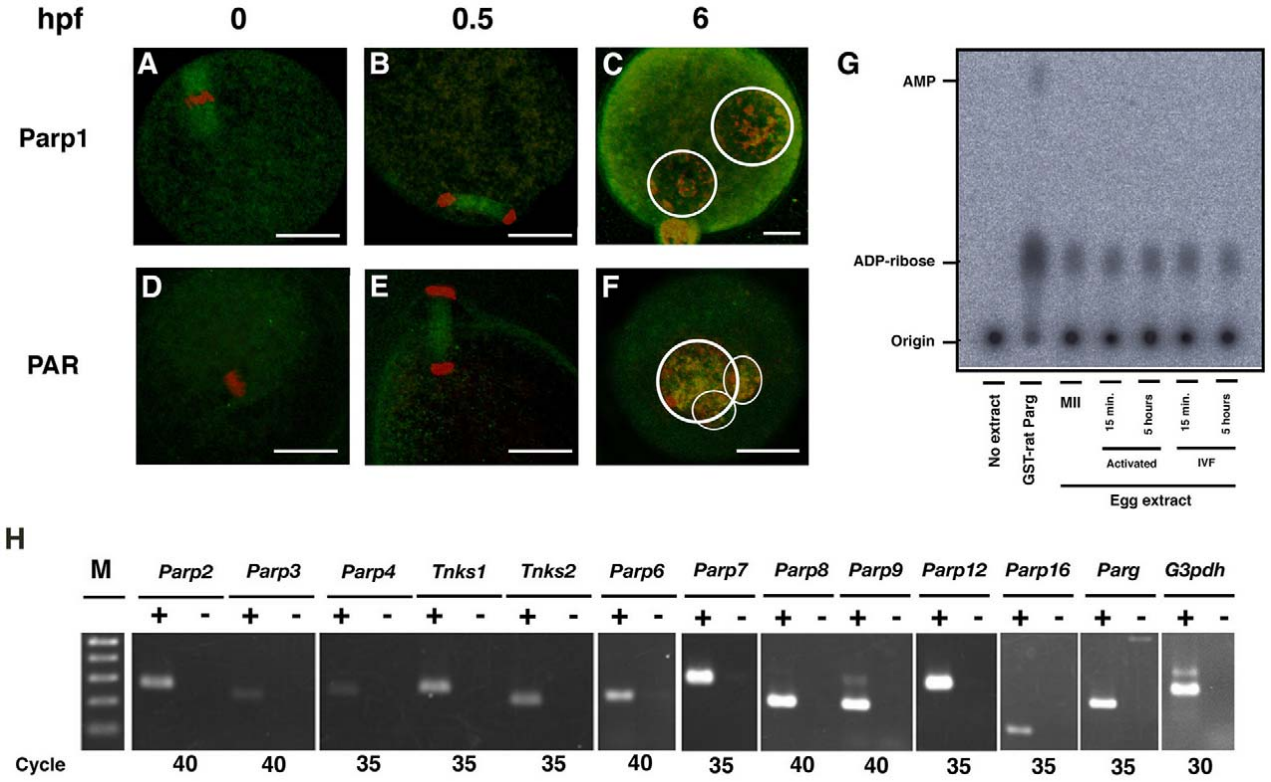
Expression of Parp, PAR level and Parg activity in the mouse oocytes. Immunofluorescence analyses of MII oocytes (**A, D**), embryos at 0.5 hpf (**B, E**) and 6 hpf (**C, F**) with antibody for Parp1 (**A–C**) and PAR (**D–F**). Detected antigens were colored with green. DNA was counterstained with PI, colored in red. White circles represent the outlines of pronuclei (PNs). Bars indicate 20 µm. (**G**) Thin layer chromatography (TLC) for the detection of poly(ADP-ribose) glycohydrolase (Parg) activity. Purified GST-Parg proteins and crude extracts from MII oocytes, parthenogenetic (activated) and untreated (IVF) embryos at 15 min and 5 hrs after activation/fertilization were loaded and reacted with synthetic PAR polymers. Release of ADP-ribose was detected by the mobility of spots from origin. A spot with high-mobility in the GST-rat Parg-loaded lane represents adenosine monophosphate (AMP). (**H**) RT-PCR analyses with primer sets for the 12 *Parp*-family genes, the *Parg* gene, and glyceraldehyde-3-phosphatase dehydrogenase (*G3pdh*) gene. Amplified DNA with cDNA synthesized with reverse transcriptase (+) or without enzymes (−) was loaded in each lane. PCR reaction was carried out with the number of cycles indicated. The 100-bp ladder marker DNA was shown (M). The lowest DNA band corresponds to the 100 base pair (bp).

To assess the presence and activation of PARylation system in oocytes, we first examined the localization of Parp1 and poly(ADP-ribose) (PAR) in the MII oocytes and one-cell embryos. Immunoreactivity on meiotic spindles of MII oocytes was detected for Parp1, but not for PAR (Figure 1A, D). Upon fertilization, signals on meiotic spindles were detected for both Parp1 and PAR (Figure 1B, E). Six hours after IVF, pronuclear staining was observed for both Parp1 and PAR (Figure 1C, F). We next analyzed Parg activity by measuring the release of ADP-ribose from PAR as substrates in the extracts prepared from MII oocytes, Sr^2+^-activated parthenogenetic embryos and IVF one-cell embryos (Figure 1G). The Parg activity was detected in all of the above, indicating that Parg also regulates PARylation in unfertilized and postfertilized (activated) eggs. The RT-PCR analyses exhibited that 12 of 17 *Parp* family and the *Parg* genes were detectable (Figure 1H).

### Developmental arrest of PJ-34 treated embryos at the pronuclear fusion during postfertilization development

**Figure 2:**
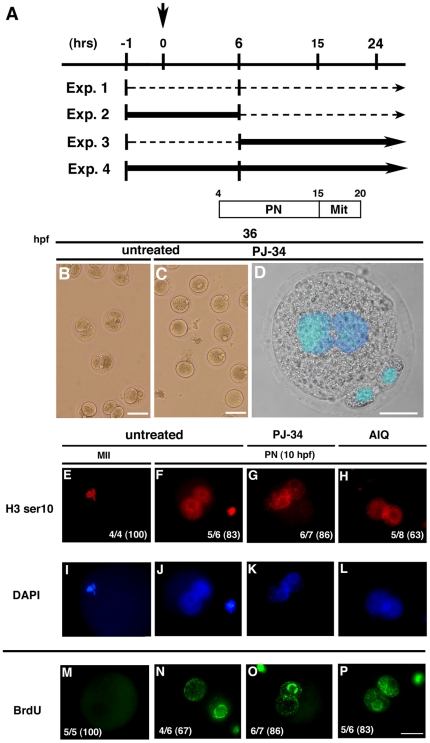
Inhibition of PNEB by PJ-34 and 5-AIQ. IVF and ICSI experiments were performed to assess the effects of Parp inhibitors at four different time-points during postfertilization development. (**A**) Scheme of the experimental design shows the incubation time of oocytes/embryos in normal culture medium (dotted line) or with Parp inhibitor treatment (bold line). The untreated IVF embryos reached the pronuclear stage (PN) approximately 4 hrs post-fertilization (hpf), and then underwent mitosis (Mit) from 15 to 20 hpf (open space) (**B**, **C**). Morphology of one-cell embryos at 36 hpf. Fertilized embryos were cultured for 36 hrs after insemination under untreated conditions (**B**) or with PJ-34 (**C**). A representative oocyte treated with PJ-34 shows defects in PNEB (**D**). DNA was counterstained with DAPI in blue. Immunofluorescence of untreated MII oocytes (MII) and PN embryos (PN), 30 µM PJ-34-treated, and 20 µM AIQ-treated PN embryos at 10 hpf with antibody for the phosphorylated form of histone H3 at serine 10 (H3 ser10, red) (**E–H**), and bromodeoxyuridine (BrdU, green) (**M–P)**. DNA of embryos reacted with H3 ser10 antibody was counterstained with DAPI in blue (**I–L**). Frequency of the staining indicated in each panel was shown in each figure (white letters). The values in parenthesis indicate percentage of the frequency. Bars represent 25 µm.

We next performed IVF to examine the significance of PARylation in postfertilization period (Table 1, Figure 2A). Effects of PARylation inhibition were assessed with 4 different protocols (Figure 2A). In Exp. 1, no Parp inhibitor was added. In Exp. 2, Parp inhibitors were added 1 hour before IVF or ICSI, subsequently incubated for 6 hrs, and then embryos were transferred in the new culture media without Parp inhibitors. In Exp. 3, Parp inhibitors were added 6 hrs after IVF or ICSI. In Exp. 4, Parp inhibitors were added 1 hr before IVF or ICSI and were present throughout the experiments. PJ-34 [14] and 5-aminoisoquinolinone (5-AIQ) [15] were used as PARylation inhibitors in this study. IC_50_ of PJ-34 for PARP-1 activity is 20 nM [14]. In normal or cancer cells, inhibitory effect of PJ-34 on PARylation is usually assessed at around 3-10 µM, for example, in A549 cells [16], and cardiac fibroblasts [17]. On the other hand, the effects of PARylation inhibition are also assessed at 30 µM PJ-34 in macrophages [18], T cells [19], neurons [20], or human breast cancer cells MCF-7, and MDA231 [21]. The permeability of the oocyte membrane is unique, and many investigations have been carried out at different concentrations of chemicals compared to the cases of cell lines *in vitro*. 3-AB was used at 5 mM as a Parp inhibitor for oocyte treatment, which has IC_50_ value for PARP-1 activity around 30 µM [22]. Based on this information we used a concentration of 30 µM PJ-34 for treatment of oocytes in this study. The first mitotic cleavage was not completed when the IVF embryos were incubated with 30 µM PJ-34 (0/189 (0%)) or 20 µM 5-AIQ (0/78 (0%), Exp. 4) (Table 1). Transition beyond the 2-cell embryos was slightly perturbed when incubated with 6 µM PJ-34 (86/90 (95.6%)) or 4 µM 5-AIQ (64/78 (82.1%)) (data not shown). The lower frequency of development to 2-cell embryos was observed when the embryos were incubated for 18 hrs from 6 hrs after IVF with 30 µM PJ-34 (4/126 (3%)) and 20 µM 5-AIQ (3/71 (4.2%), Exp. 3). We also performed intracytoplasmic injection (ICSI) experiment to omit the possible damage on sperm DNA by the PARylation inhibitors in the culture medium. Development to 2-cell embryos was stopped when the ICSI embryos were incubated for 25 hrs with either 30 µM PJ-34 (0/104 (0%)) or 6 µM PJ-34 (7/102 (7%), Exp. 4). The frequency of developmental arrest was higher in the ICSI embryos treated with 30 µM PJ-34 from 6 hrs after injection of sperm into oocytes (0/63 (0%), Exp. 3), than embryos treated with PJ-34 for 6 hrs after injection of sperm into oocytes (26/74 (35%), Exp. 2). These data indicate that PARylation inhibition results in developmental arrest of the first mitotic cleavage in mice. Experiments with PARylation inhibitors suggest that PARylation is more important for the late PN stage of mouse embryogenesis. ICSI experiments suggest that developmental defects by PARylation inhibition are mainly due to the effects on maternal genetic materials or proteins.

**Table 1:** Postfertilization development of untreated and PARP inhibitor-treated mouse embryos.

Test	Exp.*	Chemicals	Dose (µM)	No. oocytes examined	No. of embryos developed to 2-cell (%)** Blastcyst (%)**	
IVF	1	untreated	0	258	245 (100)^a^	224 (96.1)
	2	PJ-34	30	174	161 (95.8)	134 (93.3)
		5-AIQ	20	63	58 (99)	41 (92.1)
	3	PJ-34	30	126	4 (3.2)^a^	0 (0)^a^
		5-AIQ	20	71	3 (4.2)^a^	0 (0)^a^
	4	PJ-34	30	189	0 (0)^a^	0 (0)^a^
		5-AIQ	20	78	0 (0)^a^	0 (0)^a^
ICSI	1	untreated	0	118	103 (87.3)	94 (79.7)
	2	PJ-34	6	86	67 (77.9)	62 (72.1)
		5-AIQ	30	74	26 (35.1)^b^	22 (29.7)^b^
	3	PJ-34	6	67	11 (16.4)^a^	3 (4.5)
		5-AIQ	30	64	0 (0)^a^	0 (0)^a^
	4	PJ-34	6	102	7 (6.9)^a^	6 (5.9)^a^
		5-AIQ	30	104	0 (0)^a^	0 (0)^a^

*PJ-34 or 5-AIQ was added 1 hr before IVF or ICSI and removed 6 hrs later (Exp. 2), added 6 hrs after IVF or ICSI (Exp. 3), and added 1 hr before IVF or ICSI and continuously treated for subsequent 24 hrs (Exp. 4). Inhibitors were included in the medium at the designated doses and the developmental effects were shown. Both PJ-34 and 5-AIQ were used for IVF and PJ-34 was used for ICSI. Development of untreated embryos was assessed as positive control (Exp. 1). Each experimental procedure was illustrated in Figure 2A.

**Percentage is calculated by the formula: 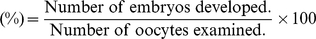.

a, b Statistic significance is compared with number of untreated oocytes or embryos by t-test (p<0.01 (a), p<0.05 (b)).

We observed a vast majority of non-treated one-cell embryos 36 hrs after IVF progressed to the first mitotic cleavage (Figure 2B), whereas PARylation-inhibited embryos stopped at the pronuclear envelope breakdown (PNEB) (Figure 2C, D). Further analyses evaluated the cell cycle progression of PARylation-inhibited embryos by immunofluorescence. BrdU incorporation (Figure 2M–P) and phosphorylation of histone H3 at serine 10, which is known as a G2 phase and mitotic marker, were detected in the both pronuclei (PNs) of one cell embryos untreated or treated with 30 µM PJ-34 or 20 µM 5-AIQ 10 hrs after IVF (Figure 2E–L). These data indicate that DNA synthesis and progression to G2 phase are occurred in both untreated or PARylation inhibited PNs.

Male PNs were preferentially stained with the phosphorylated histone H3 antibody by PARylation inhibition, which may be due to the accelerated S-phase progression of male PNs. However, there is another possibility that the increased content of histone H3 in male PNs compared to female PNs by PARylation inhibition could cause the differential staining with phosphorylated histone H3 antibody. Further analysis should elucidate the detailed roles of PARylation in the replication timing in PNs. These data suggest that PARylation is required for PNEB, but DNA synthesis and cell-cycle progression to G2 phase are not largely affected by PARylation inhibitor.

### Spindle modification by PARylation in MII oocytes and postfertilization development

**Figure 3:**
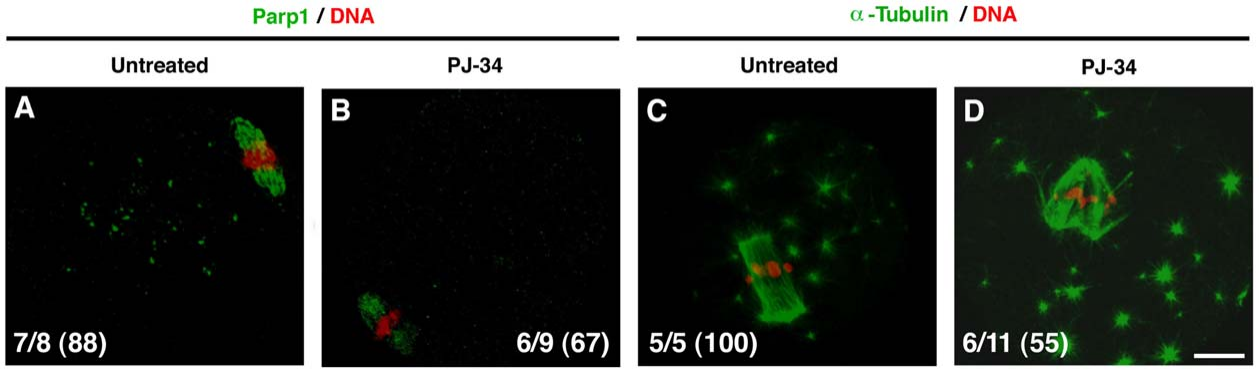
Parp1 expression, effects of PARylation inhibition on spindle bundle formation, PARylation on tubulins. (**A–D**) Immunofluorescence of untreated (**A, C**) and MII oocytes treated with 30 µM PJ-34 for 1 hr (**B, D**) with antibody for Parp1 (**A, B**) and a/ß-tubulin (**C, D**). Immunosignals were colored in green. DNA was counterstained with PI (red). Frequency of the indicated images was shown in each panel (white). Values in the parenthesis indicate the percentage as the frequency (**A–D**). Bar represents 20 µm. (**E, F**).

To explore the cause of developmental arrest by PARylation inhibition, we first focused on spindle formation, because Parp1 signals were predominantly localized to the meiotic spindles and cytoplasmic asters of MII oocytes (Figures 1A, B & 3). Administration of PJ-34 at 30 µM did not affect the localization of Parp1 at spindles (Figure 3A, B). One-hour incubation of MII oocytes in PJ-34-containing medium induced breakage of the bipolar structure of spindle bundle (untreated; 0/5 (0%), PJ-34 treated; 6/11 (55%)) with intact cytoplasmic asters (Figure 3C, D).

**Figure 4:**
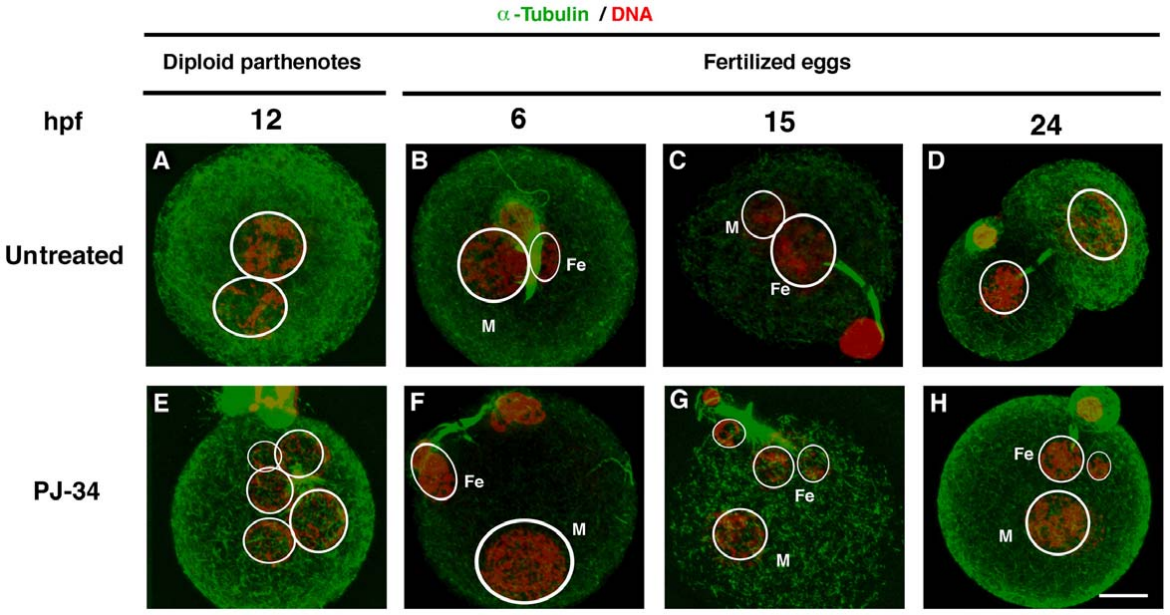
Effect of PARylation inhibition on spindle and polynucleation during postfertilization development. Immunofluorescence with a laser-scanning confocal microscopy of diploid parthenotes (**A, E**) and IVF embryos at 6 (**B, F**), 15 (**C, G**) and 24 (**D, H**) hpf. a-Tubulin signals were colored in green with untreated (**A–D**) and 30 µM PJ-34-treated (**E–H**) embryos. White circles represent the outlines of female (Fe) and male (M) pronuclei. DNA was counterstained with PI (red). Bar represents 20 µm (**H**).

During postfertilization processes, immunofluorescence for a-tubulin showed that spindle between female PNs and the nucleus of the second polar body and cytoplasmic tubulins were similarly detected in both untreated and PJ-34 treated embryos (Figure 4A–H). We noticed that polynuclei of PJ-34 treated embryos were frequently detected (untreated; 2/21 (10%), PJ-34; 11/24 (45.8%), *p*<0.01). Most nuclei were connected with maternal spindles and single paternal nuclei were segregated (Figure 4B, C, F, G). These data suggest that polynuclei come from maternal genetic materials.

### Identification of PARylation of a-tubulin and a defect in Erk activation in *Parp1^−/−^* oocytes

Because a-tubulin dynamics were affected by Parp inhibitors, we investigated whether a-tubulin is PARylated during the fertilization processes. For this purpose, we carried out two-dimensional gel (2D) electrophoresis followed by western blots and MALDI-TOF mass spectrometry. Western blots with the 2D electrophoresis analysis exhibited that several molecules including tubulins (a1c, §2c) were PARylated in the untreated MII oocytes (Figure 3). PARylation is suggested to be important for tubulin dynamism in the MII oocytes and a/ß-tubulins could be the potential target for PARylation.

**Figure 5:**
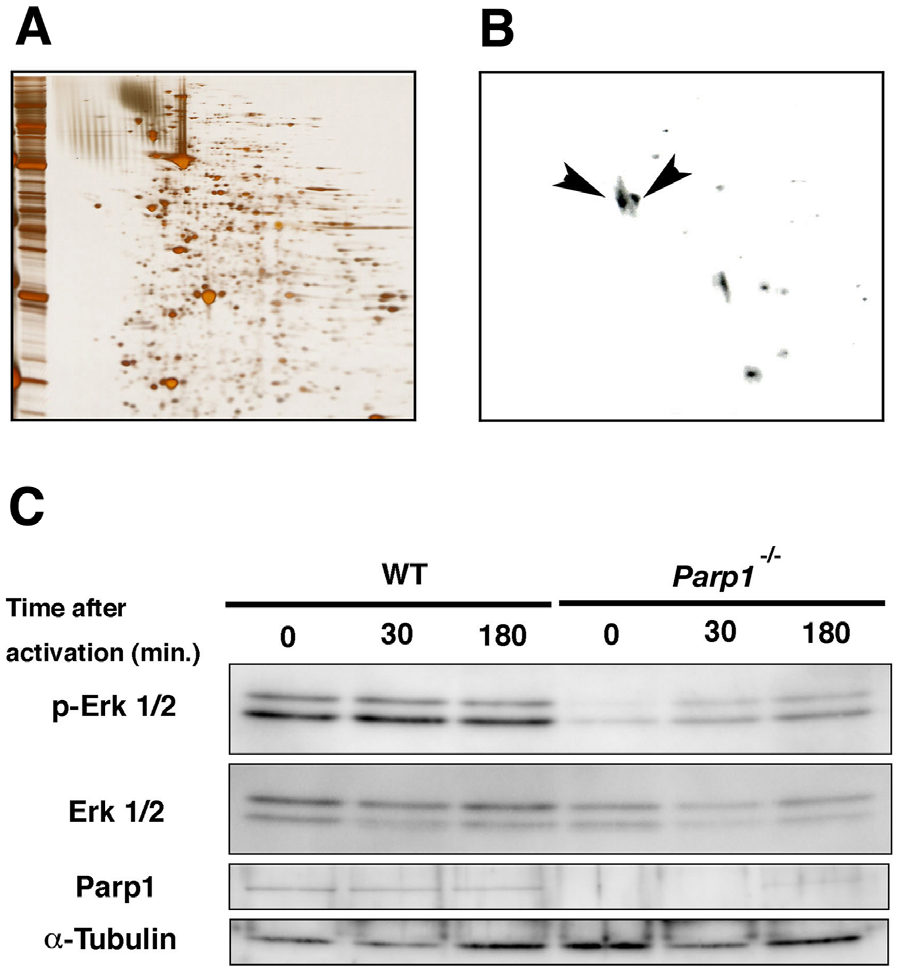
Biochemical analyses of PARylated proteins of MII oocytes and effects of Parp1 on the Erk-signaling. The 2D electrophoresis of 300 untreated MII oocytes with silver staining (**A**) and immunoblots with the monoclonal antibody for poly(ADP-ribose) polymers (clone 10H) (**B**). The protein spots corresponding to the immunosignals were identified as a1c (left arrowhead) and β2c (right arrowhead) tubulins by MALD-TOF mass spectrometry. (**C**) Western blotting of the protein extracts of MII oocytes of wild-type (WT) and *Parp1^−/−^* mice was performed with antibodies against the phosphorylated form of Erks (p-Erk 1/2), Erks, pan (Erk 1/2), Parp1, and a-tubulin as a control (**C**). The oocytes were collected 30 and 180 minutes after the activation of MII oocytes with Sr^2+^. Proteins loaded in each lane corresponded to 30 oocytes.

Inhibition of the Erk phosphorylation leads to defective microtubule organization [23]. PARP1 is also reported to interact with Erk-1/2 [24], [25]. Therefore, we investigated the activation of Erk 1/2 in the *Parp1^−/−^* MII oocytes. Phosphorylation of Erk-1/2 was down-regulated in the MII oocytes of *Parp1^−/−^* oocytes, compared with *Parp1^+/+^* oocytes, while total amount of Erk-1/2 was comparable between the genotypes (Figure 5C). However, a low level of phosphorylation of Erk is still upregulated after activation in a Parp1-independent manner. Collectively, PARylation may be important for the integrity of meiotic spindle formation mediated in part by phosphorylation regulation of Erk by Parp1 in the MII oocytes and PARylation on tubulins in the postfertilized one-cell embryos.

### Defective phosphorylation of lamin by PARylation inhibition

We examined the chromosomal behavior at the late PN stage by the DNA-staining, because the pronuclear envelope was still detected in the PARylation-inhibited embryos. Condensation of chromatin was detected in non-treated one-cell embryos, whereas it was undetected in the PJ-34 treated embryos at 15 hpf stained by 4′,6-diamidino-2-phenylindole (DAPI) or propidium iodide (PI) (untreated, 28 cells in prometaphase/78 cells examined (35.9%); PJ-34 treated, 0 cells/94 cells (0%)).

**Figure 6:**
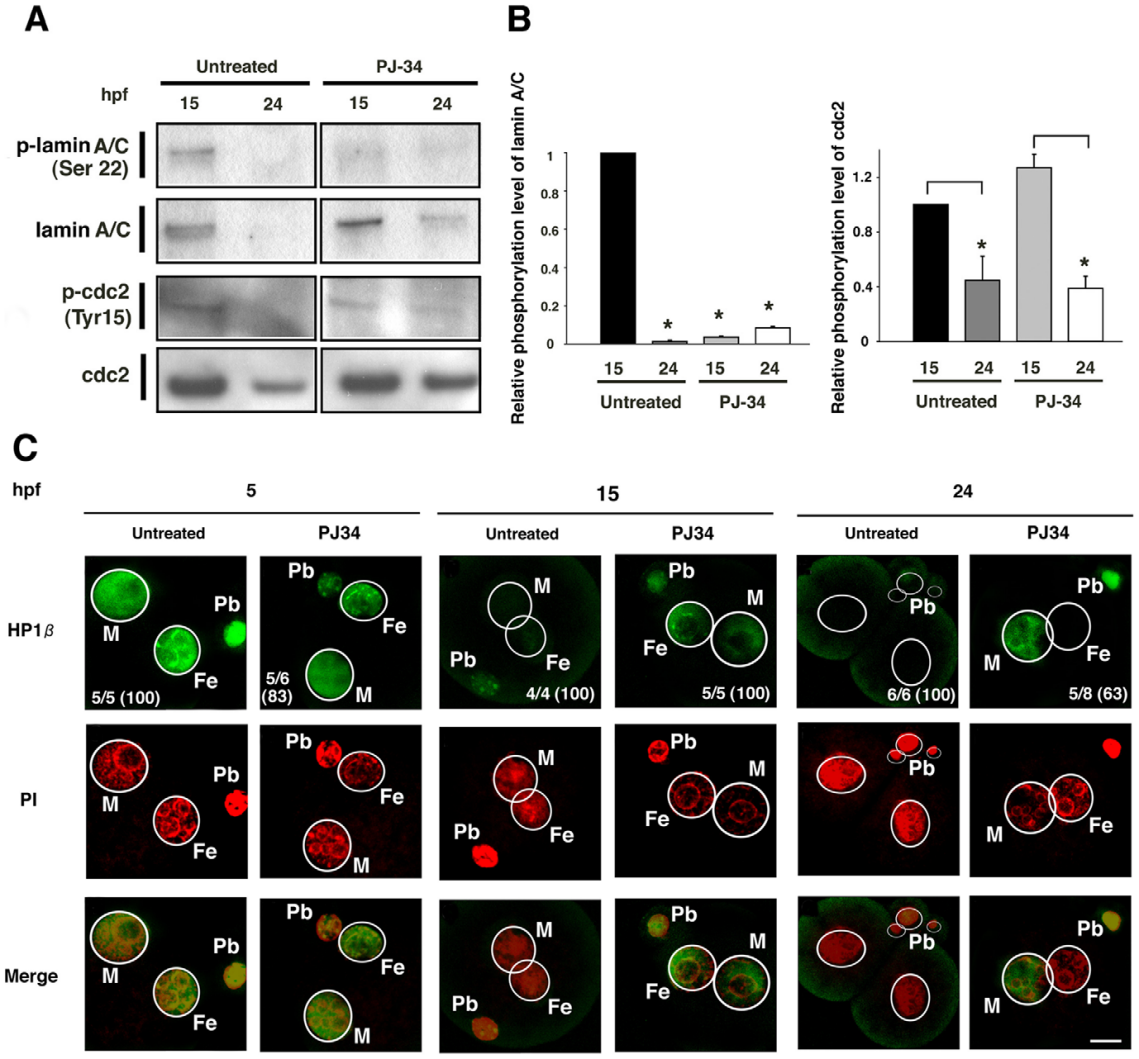
Defective phosphorylation of pronuclear lamins and behavior of HP1β during postfertilization development. Extracts from 100 untreated or PARylation inhibited embryos at 15 and 24 hpf were immunoblotted with antibody for phosphorylated lamin A/C, total lamin A/C, phosphorylated cdc-2, and total cdc-2 (**A**). Assessments for the relative phosphorylation levels of lamin and cdc2. Asterisks represent statistic significance (t test, p<0.05) (**B**). Immunofluorescence with laser-scanning confocal microscopy of untreated and PJ-34 treated embryos with antibodies for HP1β (**C**). Detected antigens were colored with green. DNA counterstained with PI was colored in red. Colocalized signals of antigens and DNA were colored in orange or yellow in merged figures. Circles (white lines) show the outlines of the female (Fe) and male (M) PNs. Other PI signals represent polar bodies (Pb). Values (percentage in parenthesis) represent frequency of the staining in each panel (**C**, upper panels). Bar represents 25 µm.

Nuclear envelope breakdown is initiated by the phosphorylation of lamins [26]. We found that the lamin A and C appear to be components of the pronuclear lamina of embryos and PJ-34 treatment blocks phosphorylation of lamin A/C at 15 hpf (Figure 6A, B). Lamin A/C was largely undetectable in untreated embryos at 24 hpf, because the amount of maternal lamin A/C was reduced after the first mitosis [27]. In contrast, lamin A/C was persistently detectable in the PJ-34 treated embryos at 24 hpf, while phosphorylation of lamins was not detected (Figure 6A, B). The cdc2 kinase activity is responsible for phosphorylation of lamins [28]. Western blots revealed that between 15 hpf and 24 hpf, the phosphorylation of tyrosine 15 (Tyr15) of cdc2 (p-cdc2) was reduced in both untreated and PJ-34-treated embryos (Figure 6A). Immunocytochemistry revealed the p-cdc2 signals in the PNs of untreated and PJ-34 treated embryos (data not shown). These data suggest that the inhibition of lamin A/C phosphorylation by Parp inhibitor was not caused by affecting the activity of cdc2 kinase.

As a lamina-associated protein interacting with Parp1 [29], we examined the behavior of heterochromodomain protein 1ß (HP1ß) by immunofluorescence. A broad staining pattern of HP1ß was observed in the PNs of untreated embryos (Figure 6C). This staining pattern was reported in a previous study [30]. At 15 hpf, HP1ß signals became absent in untreated PN embryos, while a persistent localization of HP1ß was detected in PJ-34 treated embryos (Figure 6C). HP1ß was still detectable only in male PNs of one cell embryos by PARylation inhibition, but undetectable at the nuclei of two-cell embryos in 24 hpf embryos in the absence of PJ-34 treatment (Figure 6C). This data implies that PJ-34 delayed the HP1ß removal from chromatin of PNs and this could be involved in attenuated phosphorylation of lamin A/C.

## Discussion

**Figure 7:**
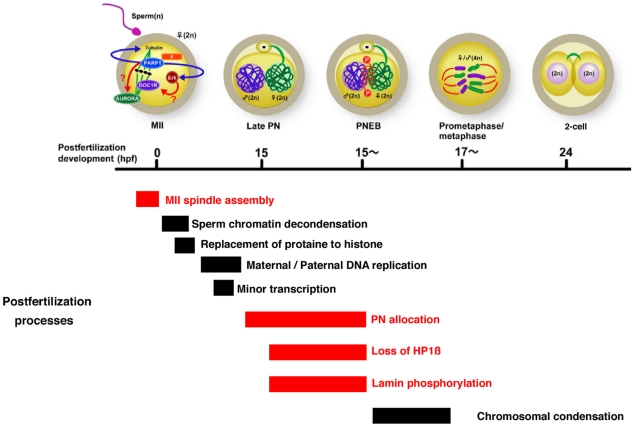
Scheme of spacio-temporal regulation by PARylation during perifertilization mouse development. At MII oocytes, Parp1 or other Parps are involved in spindle bundle formation mediated in part by Erk phosphorylation, which is represented as MII spindle assembly. Other Parp-associated molecules (Aurora [47]) or MAPK-associated molecules such as DOC1R [48] putatively contribute to spindle formation integrity. HP1ß loss from PNs and PN allocation are interfered by Parp inhibitors during PN stages. Phosphorylation of lamin A/C is reduced by PARylation inhibition, and subsequently PNEB is blocked. Because HP1β is associated with both chromatin and nuclear envelope, both chromatin and nuclear envelope are putatively regulated by PARylation. Duration of biological processes during 24 hrs after fertilization (open square) is listed temporally and those marked in red have been suggested to be regulated by PARylation from this study.

Our data from studies by pharmacological inhibition of PARylation in mice suggests that PARylation has three major functions in peri-fertilization processes (Figure 7). First, PARylation affects proper spindle formation in the mouse MII oocytes. Our data supports the view of Parp function described in a previous study using *Xenopus* oocytes [31]. The a/§-tubulins, which have been reported as major components of microtubules, are shown to be acceptor proteins for PARylation in mouse oocytes [32]. Our data suggest that PARylation may regulate the meiotic spindle assembly of MII oocytes either directly through PTM of tubulin, indirectly by altering MAPK signaling, or both. Immunofluorescence study showed no specific localization of PAR signals in MII-oocytes, while spindle bundle formation is perturbed by PARylation inhibition. Cytoplasmic tubulins are broadly present in mouse oocytes and associated with Erk and kinesins. Cytoplasmic Parp1 is associated with microtubule mediated by kinesins and modulates Erk signaling in neurons [24], [25], [33]. Our data shows downregulation of phosphorylation of Erk-1/2 in *Parp1^−/−^* MII oocytes. Collectively, we speculate that Parp1 may act as an activator of Erk-1/2 phosphorylation in the MII oocytes. Spindle bundle formation could be also regulated by other tubulin-associated Parps (Figure 6). Related to this view, interaction of tankyrase-1 with NuMA as an acceptor protein of poly(ADP-ribose) was reported to regulate mitotic spindle assembly [34]. Phosphorylation of Erks is downregulated in *Parp1^−/−^* eggs before and after activation. However, it seems that a low level of Parp1-independent phosphorylation of Erk is induced after activation, although Erk signaling is gradually inactivated upon fertilization in normal development. The PAR signals induced on meiotic spindles for a brief period during postfertilization development suggest that spindle-associated Parp1 is activated, that could mediate the Erk-signaling upon fertilization.

Intracytoplasmic Ca^2+^ release stimulates the NAD metabolism after binding of sperm to the egg cell membrane [35]. Parp catalyzes PARylation of proteins using NAD with production of nicotinamide. Thus, we speculate a novel possibility that PARylation functions as a mediator of NAD signals upon fertilization.

Second, the inhibition of PARylation results in the complete inhibition of PNEB. Mechanism of PNEB remains largely unknown. The phosphorylation of nuclear lamins is a major process in PNEB. Here, we showed that the phosphorylation of lamins A and C is hampered by the inhibition of PARylation. Interestingly, although the cdc2 kinase activity is responsible for the phosphorylation of the lamin meshwork, our data indicates that these activities are intact in PJ-34-treated embryos. Lamin A negatively affects epigenetic regulation of the myogenin gene expression in mouse myoblasts [36]. Synthesis of lamin A is absent during the cleavage-stage blastomere [37] and in stem cells [38]. Recently, Parp1 was found to co-localize with HP1 that promotes the interaction of heterochromatin with the inner layer of the nuclear envelope [29]. We found that, in contrast to untreated embryos, HP1ß signals were persistently detected in the male PNs of PJ-34-treated embryos at 24 hpf. Together with the evidence from a previous study describing that PARylation also functions in telomere regulation [39], our data raise the possibility that PARylation functions as a regulatory machinery of the pronuclear envelope disassembly.

Our data using PJ-34 and 5-AIQ was different from those obtained with 3-aminobenzamide (3-AB) [22]. We found that IVF experiments and *in vitro* culture of one-cell embryos with 5 mM 3-AB did not show any influence on the transition of the first mitotic cleavage. In this study, we did not observe the inhibition of PAR activation at the PN stage by 5 mM 3-AB (data not shown). Our immunofluorescence showed residual signals of PAR at the PNs of embryos treated with 5 mM 3-AB, but barely with PJ-34 and 5-AIQ. Collectively, we found that PJ-34 or 5-AIQ, water-soluble PARP inhibitors, are more suitable for a more precise assessment of inhibition of total PARylation activity at postfertilization development. Further analyses will be needed to seek for the functional PARPs, which regulate MII spindle assembly, signal transduction of PNEB processes during early stages of postfertilization development.

Recently, PARylation inhibitors emerge as effective therapeutic agents for mammary tumors [40]. Our study also implicates a risk of subfertility by administrating PARP inhibitors. Furthermore this study raises a possibility of PARP inhibitors as potent contraceptive chemicals. Molecular dissection of the PARylation system will provide our understanding for significance of PTM in mammalian development [41].

## Materials and Methods

### Oocyte and embryo manipulations

*Parp1^−/−^* mutant mice under a B6D2F1 hybrid background were generated by sequential backcrosses of *Parp1^+/−^* mice to C57BL/6J and DBA2/J [11]. Oocytes were collected from superovulated B6D2F1 females 14 hrs after the intraperitoneal injection of equine chorionic gonadotropin (eCG), followed by the injection of human chorionic gonadotropin (hCG). For IVF, oocytes were cultured in TYH medium [42]. Sperm was collected from the caudal epididymis of B6D2F1 males and incubated in TYH medium. After the preincubation of MII oocytes in 200/300 µL of TYH medium with or without PJ-34 or 5-AIQ as described in Table 1, sperm (150 sperm/µL) was added to the oocytes. Intracytoplasmic sperm injection (ICSI) was carried out essentially as described previously [43]. ICSI was carried out in Hepes-buffered CZB medium with or without 30 µM PJ-34. Thereafter, embryos were cultured in modified Whitten medium [44].

### Animal care

All animals were housed according to the institutional guidelines in compliance with National Institutes of Health guidelines. Experiments using animals were approved by the Animal Care and Use committee of Mitsubishi Kagaku Institute of Life Sciences, MITILS, and National Cancer Center Research Institute (T05-026-MB06, T05-026-CB07).

### Antibodies

The antibodies and dilutions used in this study were described in Supplemental Table S1. The HRP–conjugated rat or rabbit IgG (1∶10000, Jackson Immunoresearch Laboratory) and HRP-conjugated mouse IgG (1∶1000, Bio-Rad) as the secondary antibodies for immunodetection and the Alex Fluor conjugates (1∶200, Invitrogen) of IgG for immunofluorescence.

### Immunofluorescence

The cumulus-oocyte complex was dissociated by hyaluronidase (Sigma) and the zona pellucida was removed with 0.5% actinase (Kaken, Japan). After incubation of the denuded oocytes or embryos for at least 30 minutes, the oocytes or embryos were placed into fibrin clots [45]. The fibrin clots were prepared by mixing 1 µl fibrinogen (12.5 mg/ml PBS: Calbiochem) with 1 µl thrombin (10 mg/ml distilled H_2_O: Sigma). For the staining of microtubules, oocytes and embryos were permeabilized by microtubule stabilizing buffer (60 mM PIPES, 25 mM HEPES, 10 mM EGTA, 2 mM MgCl_2_, adjusted to pH 6.9) for 3 minutes, and then fixed with 3% formaldehyde for 10 minutes at 37°C. The cells were incubated with primary antibodies for 1 hr or overnight at 37°C, followed by incubation with blocking buffer (PBS containing 5% normal goat serum and 0.05% Tween-20). The cells were then incubated with secondary antibodies for 1–2 hrs at 37°C. After washing with PBS, the cells were counterstained with PI and subjected to microscopic analyses. For the staining with histone modification antibodies, the cells were fixed with 4% paraformaldehyde for 15 minutes at 4°C and then permeabilized with PBS containing 0.1% BSA and 0.5% TritonX-100 for 10 minutes. The cells were incubated with primary antibodies overnight at 4°C, and then with secondary antibodies for at least 3 hrs at 4°C. For detection of DNA synthesis, the IVF embryos at 8 hpf were transferred to Whitten medium containing 10 µM 5-bromo-2′-deoxyuridine (BrdU, Roche Diagnostic Corporation) and incubated for 2 hrs. Embryos were then fixed and permeabilized as described above. After washing for three times, embryos were incubated in culture medium containing 2 N HCl for 30 min. Embryos were washed for 5 times with borate buffer (pH 8.5) and then for 3 times with PBS. Neutralized embryos were incubated for 30 min with PBS containing 5% FBS, and then reacted with anti-bromodeoxyuridine monoclonal antibody (Roche Diagnostic Corporation) overnight at 4°C. The images were captured under a light microscopy (Axiophot, Zeiss) or confocal microscopy (IX71 with Fluoview FV300, Olympus) system.

### Immunoblots

For the two-dimensional gel electrophoresis, oocyte extracts were collected into sample buffer (7 M urea, 2 M thio-urea, 4% CHAPS, 40 mM DTT, 2% IPG buffer (pH 3–10) (GE Healthcare), protease inhibitor cocktail). Samples were purified using the 2D Clean-UP kit (GE Healthcare). Protein extracts corresponding to 300 MII oocytes or one-cell embryos were applied to an Immobiline drystrip (pH 3–10, GE Healthcare) and subjected to isoelectric focusing with a multiphor apparatus (GE Healthcare) as the first protein separation. The stripe was then applied to a 10% SDS-PAGE gel for the second protein separation. The proteins on the gels were transferred to a BioTrace nitrocellulose membrane for histones WB (pore size, 0.2 µm, Pall) or Immobilon PVDF membrane (Millipore), and reacted with primary antibodies for 1 hr at room temperature. The membrane was washed and then reacted with secondary HRP-conjugated antibodies (see below). The signals were enhanced by Can Get Signal, an immunoreaction enhancer solution (Toyobo) and detected with the Chemi-Lumi One kit (Nacalai) and Amersham Hyperfine ECL (GE Healthcare).

### Parg analysis

Eggs in the Parg buffer (20 mM potassium phosphate (pH 7.5), 0.1% Triton X-100, 10 mM §-mercaptoethanol) were mixed for 30 minutes at 4°C by a mixer, and then subjected to freezing and thawing five times. Aliquots of the extracts corresponding to 2 eggs were used for the thin layer chromatography (TLC) assay. Fifteen nanograms of recombinant GST-Parg (0.3 µL) were used as control. Extracts were incubated for 2 hrs at 25°C with ^32^P-labeled PAR. The reactions were terminated by adding 0.1% SDS. The samples were spotted on a polyethylene impregnated cellulose TLC plate (Macherht-Nagal), developed with a developing buffer [46], and then detected by BAS2500 (Fuji Film).

### RT-PCR

Total RNA was isolated from 100–150 MII oocytes using Sepasol-RNA I Super (Nacalai). The cDNA was synthesized by Superscript III (Invitrogen) according to the manufacturer's instructions. The PCR reactions were carried out at 55 and 60°C as the annealing temperature with 30 and 40 cycles. The primer sequences used in this study and expected length of amplified cDNA fragments were described in Table S2.

## Supporting Information

Table S1 The primary antibodies used in this study.
(0.13 MB TIF)

Table S2 The primer sequences to detect *Parps*, *Parg*, and *G3pdh* genes by RT-PCR.
(9.44 MB TIF)
