# Supplementary figures and images for: PolyADP-Ribosylation Is Required for Pronuclear Fusion during Postfertilization in Mice
Source: PLoS One. 2010 Sep 2;5(9):e12526. doi: 10.1371/journal.pone.0012526 (PMC2932744; doi:10.1371/journal.pone.0012526)

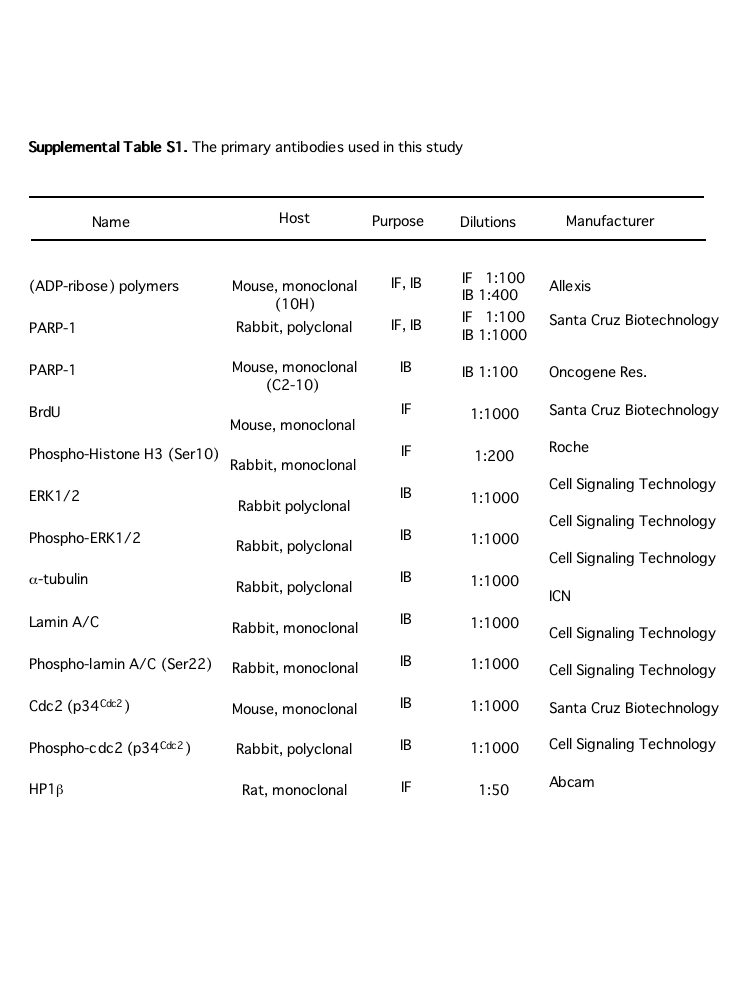

Supplement: Table S1 — The primary antibodies used in this study. (0.13 MB TIF) [file pone.0012526.s001.tif]

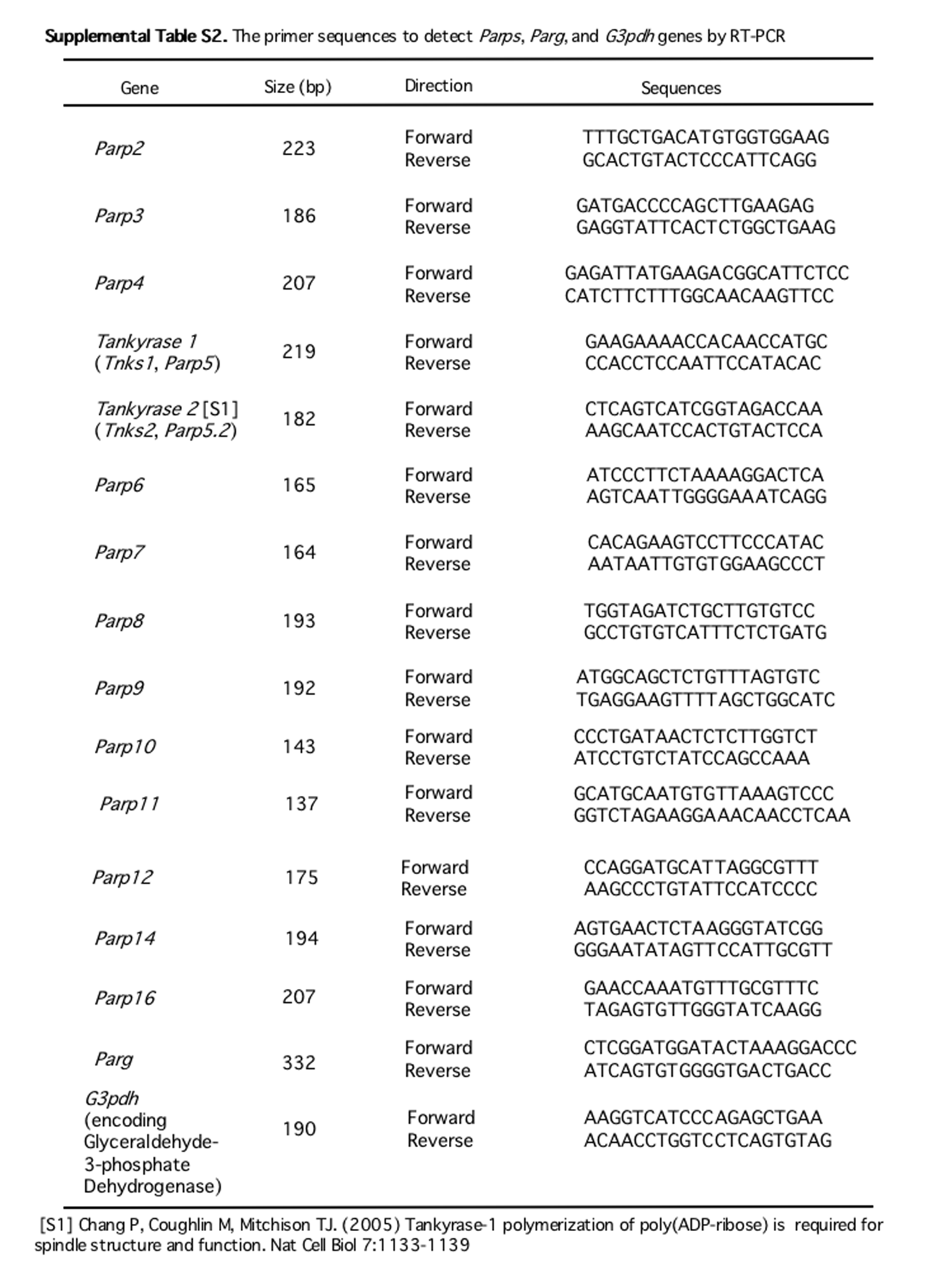

Supplement: Table S2 — The primer sequences to detect Parps, Parg, and G3pdh genes by RT-PCR. (9.44 MB TIF) [file pone.0012526.s002.tif]
